# Integrative RNA, miRNA, and 16S rRNA sequencing reveals immune-related regulation network for glycinin-induced enteritis in hybrid yellow catfish, *Pelteobagrus fulvidraco* ♀ × *Pelteobagrus vachelli* ♂

**DOI:** 10.3389/fimmu.2024.1475195

**Published:** 2025-01-15

**Authors:** Linyuan Yi, Aijie Mo, Huijun Yang, Yifan Yang, Qian Xu, Yongchao Yuan

**Affiliations:** ^1^ College of Fisheries, Key Lab of Freshwater Animal Breeding, Ministry of Agriculture, Huazhong Agricultural University, Wuhan, Hubei, China; ^2^ Shuangshui Shuanglu Institute, Huazhong Agricultural University, Wuhan, China; ^3^ National Demonstration Center for Experimental Aquaculture Education, Huazhong Agricultural University, Wuhan, China

**Keywords:** hybrid yellow catfish, glycinin, foodborne enteritis, intestinal immunity, intestinal microbiata

## Abstract

Glycinin-induced foodborne enteritis is a significant obstacle that hinders the healthy development of the aquatic industry. Glycinin causes growth retardation and intestinal damage in hybrid yellow catfish (*Pelteobagrus fulvidraco* ♀ × *Pelteobagrus vachelli* ♂), but its immune mechanisms are largely unknown. In the current study, five experimental diets containing 0% (CK), 1.74% (G2), 3.57% (G4), 5.45% (G6), and 7.27% (G8) immunological activity of glycinin were fed to juvenile hybrid yellow catfish to reveal the mechanism of the intestinal immune response to glycinin through RNA and microRNA (miRNA) sequencing and to explore the interrelation between immune molecules and intestinal microbiota. The results demonstrated that glycinin content in the posterior intestine increased significantly and linearly with the rise of dietary glycinin levels. More than 5.45% of dietary glycinin significantly reduced the nutritional digestion and absorption function of the posterior intestine. Notably, an obvious alteration in the expression levels of inflammatory genes (*tnf-α*, *il-1β*, *il-15*, and *tgf-β1*) of the posterior intestine was observed when dietary glycinin exceeded 3.57%. Sequencing results of RNA and miRNA deciphered 4,246 differentially expressed genes (DEGs) and 28 differentially expressed miRNAs (DEmiRNAs) between the CK and G6 groups. Furthermore, enrichment analysis of DEGs and DEmiRNA target genes exhibited significant responses of the MAPK, NF-κB, and WNT pathways following experimental fish exposure to 5.45% dietary glycinin. Additionally, at the level of 3.57% in the diet, glycinin obviously inhibited the increase of microbiota, especially potential probiotics such as *Ruminococcus bromii*, *Bacteroides plebeius*, *Faecalibacterium prausnitzii*, and *Clostridium clostridioforme*. In sum, 5.45% dietary glycinin through the MAPK/NF-κB/WNT pathway induces enteritis, and inflammatory conditions could disrupt micro-ecological equilibrium through miRNA secreted by the host in hybrid yellow catfish. This study constitutes a comprehensive transcriptional perspective of how intestinal immunity responds to excessive glycinin in fish intestines.

## 1 Introduction

The substitution of fish meal with plant protein in aquafeeds leads to intestinal inflammation and growth inhibition, which poses major obstacles hindering the progress of the aquaculture sector. Soybean meal has emerged as the predominant alternative plant protein source to fish meal in aquafeeds, owing to its abundant yield, elevated protein content, and reliable availability (1). However, soybean antigenic proteins, as the main anti-nutritional factor with significant abundance, resistance to heat and strong immunogenicity in soybean meal, and prolonged and increased consumption can predispose aquatic organisms to allergic reactions in their gastrointestinal tracts, culminating in intestinal damage and related complications (2, 3). As one of the most important soybean antigen proteins, glycinin accounts for nearly 41.9% of soybean protein (4), possesses an extremely compact molecular structure, and is difficult to enzymatically hydrolyze (5). Recent research reported that 3.08%–6.04% immunologically active glycinin in the diet caused intestinal inflammation and oxidative damage, destroyed immune and digestive functions, and ultimately hindered the growth of *Rhynchocypris lagowskii* Dybowski (6). Accumulating lines of evidence suggest that excessive dietary glycinin could increase intestinal mucosal permeability, trigger intestinal inflammatory pathology, or disrupt microbial balance in various aquatic animals such as turbot (*Scophthalmus maximus* L) (7), Chinese mitten crabs (*Eriocheir sinensis*) (8), grass carp (*Ctenopharyngodon idella*) (9), and orange-spotted grouper (*Epinephelus coioides*) (10).

As the largest immune organ, the intestine accompanying the strongest mucosal immune system plays a crucial role in preserving host immune homeostasis and preventing pathological immune responses (11). However, owing to the fragile digestive tract of juvenile animals, a small proportion of glycinin that has not been fully digested maintains macromolecular activity and directly crosses the intestinal mucosal barrier, thereby stimulating the immune response in the blood, lymph, and intestine, causing inflammatory tissue damage (12, 13). It is a remarkable fact that previous studies on grass carp (9, 12) and *R. lagowskii* Dybowski (6) revealed that inflammation in the posterior intestine caused by glycinin appears more serious compared with that in the anterior and mid intestines, attributed to reasons such as that epithelial cells of the posterior intestine are more sensitive to antigen binding, leading to the strongest inflammatory response (14). The resistance of fish growth to the nutrient composition and immune status may form an intestinal environment conducive to the reproduction of potentially pathogenic bacteria, thereby impacting the homeostasis of the intestinal microbial community (15). Although certain studies have reported that 8% or 10% dietary glycinin disturbs the micro-ecological equilibrium of the intestine in aquatic animals (8, 10, 16), the specific action mechanism of this process and the relationship between microbes and intestinal inflammation still have many limitations, and more explorations are needed to enrich the understanding of this active field.

New omics technologies, including RNA sequencing (RNA-seq) and microRNA sequencing (microRNA-seq), hold notable potential to explore and interpret the complex relationship between fish nutrition and immunity (17). Ambient changes can trigger changes in transcriptional expression patterns (18). Nevertheless, to date, the effects of glycinin on the intestinal transcriptome and immunomodulatory networks have not been reported, and further research is needed. MicroRNAs (miRNAs), endogenous ~22-nucleotide non-coding RNAs, directly inhibit the gene expression via mRNA cleavage and/or translation (19). MiRNAs play a cardinal role in regulating pathological processes, and their dysregulation is connected with many diseases, including inflammation (20). It is worth noting that gradually increasing studies indicated that miRNAs are cardinal communication mediators of host–microbe interactions (21–23). Host-derived miRNAs are transferred into intestinal bacteria through extracellular vesicles, modulating their gene expression and affecting the replication of intestinal microbiota (21, 24). Conversely, the microbiota can alter host miRNA expression to promote epithelial proliferation and regulate its permeability, affecting intestinal homeostasis (25). Despite that the importance of this interaction is continuously emerging, the response mechanism between miRNA and intestinal microbiota in glycinin-induced enteritis remains elusive in fish.

Hybrid yellow catfish (*Pelteobagrus fulvidraco* ♀ × *Pelteobagrus vachelli* ♂) is a new species with fast growth, strong disease resistance, high quality, and market acceptance (26). In spite of this, cultured hybrid yellow catfish frequently cause foodborne enteritis attributed to its vigorous ingestion, which causes huge economic losses to the aquaculture industry. Our previous study found that more than 5.45% dietary glycinin eminently reduced the growth performance, accompanied by the aggravation of intestinal oxidative stress and apoptosis, and the impairment of intestinal structural integrity (27). Information on how glycinin causes gastrointestinal inflammation of hybrid yellow catfish is still unclear. Hence, the aim of this research was to use omics technology to explore the immunomodulatory networks and microbial imbalance mechanism of glycinin-induced foodborne enteritis and to provide essential insight into the breakthrough of intestinal health disorders in fish.

## 2 Materials and methods

### 2.1 Diet production and breeding trial

Glycinin was isolated from soybeans obtained from Wuhan Alpha Agri-tech Co., Ltd., and the specific extraction method was the same as that described in Yi et al. (27). Additionally, this research utilized the diet preparation and growth experiment in our previous study (27). Briefly, five different levels of glycinin diets were created based on the nutritional needs of the hybrid yellow catfish being studied (**Table 1**). They included 0%, 2.08%, 4.16%, 6.24%, and 8.32% glycinin, which corresponded to substituting 0%, 20%, 40%, 60%, and 80% fish meal proteins with soybean meal proteins, recording as the CK, G2, G4, G6, and G8 groups, respectively. Raw materials were provided by Wuhan Aohua Technology Co., Ltd. (Wuhan, China). The method of feed production was described in our previous study (27). Immunological activities of dietary glycinin in the groups of CK, G2, G4, G6, and G8 were consistent with those of our previous experiment, which were 0%, 1.74%, 3.57%, 5.45%, and 7.27%, respectively (27).

**Table 1:** Formulation and nutritional content of experimental feeds (air-dry basis, %).

Components	Experimental diets				
	CK	G2	G4	G6	G8
White fishmeal	40.00	32.00	24.00	16.00	8.00
Chicken powder	10.00	10.00	10.00	10.00	10.00
Corn gluten flour	8.00	8.00	8.00	8.00	8.00
Glycinin	0.00	2.08	4.16	6.24	8.32
Casein	0.00	2.72	5.44	8.16	10.88
Soybean phospholipid oil	5.00	5.60	6.20	6.80	7.40
High gluten flour	27.50	30.10	32.70	35.30	37.90
Choline chloride	1.00	1.00	1.00	1.00	1.00
Vitamin premix ^1^	2.00	2.00	2.00	2.00	2.00
Mineral premix ^2^	2.00	2.00	2.00	2.00	2.00
Sodium alginate	2.00	2.00	2.00	2.00	2.00
Antioxidant	0.50	0.50	0.50	0.50	0.50
Anti-mildew agent	0.50	0.50	0.50	0.50	0.50
Betaine	1.00	1.00	1.00	1.00	1.00
Chromium trioxide	0.50	0.50	0.50	0.50	0.50
Nutritional composition					
Moisture	12.59	12.96	12.51	12.92	12.88
Crude protein	42.41	42.19	42.39	42.17	42.62
Crude lipid	9.23	9.14	9.73	9.13	9.38
Ash	8.80	8.92	8.71	8.30	8.14

^1^Vitamin premix (mg/kg diet): vitamin A, 1.67; vitamin D, 0.027; vitamin E, 50.20; vitamin K, 11.10; vitamin C, 100.50; folic acid, 5.20; calcium pantothenate, 50.20; inositol, 100.50; niacin, 100.50; biotin, 0.12; cellulose, 645.25.

^2^Mineral premix (mg/kg diet): NaCl, 500.15; MgSO_4_·7H_2_O, 8,155.55; NaH_2_PO_4_·2H_2_O, 12,500.51; KH_2_PO_4_, 16,000.51; Ca(H_2_PO_4_)_2_·H_2_O, 7,650.50; FeSO_4_·7H_2_O, 2,286.15; C_6_H_10_CaO_6_·5H_2_O, 1,750.12; ZnSO_4_·7H_2_O, 178.12; MnSO_4_·H_2_O, 61.35; CuSO_4_·5H_2_O, 15.45; CoSO_4_·7H_2_O, 0.89; KI, 1.5; Na_2_SeO_3_, 0.59.

Juvenile hybrid yellow catfish (1.02 ± 0.01 g) were from Hubei Huangyouyuan Fisheries Development Co., Ltd. (Wuhan, China). The breeding trial took place in the Huazhong Agricultural University aquaculture base. A total of 450 fish were randomly placed into 15 glass tanks (1.20 × 0.60 × 0.45 m) at 30 fish per tank. Experimental fish were temporarily fed with the control group feed for 2 weeks to adapt to the experimental environment. Then, five experimental diets were randomly divided into triplicate tanks. During the rearing period, the feeding management and water quality testing methods for fish were the same as those in our previous study (27).

### 2.2 Sample collection

In the eighth week, three fish were chosen at random from each group to be anesthetized using 100 mg/L MS-222 (tricaine methanesulfonate, Sigma, St. Louis, MO, USA) solutions. Subsequently, the intestinal contents were separated to detect intestinal microbes. Six fish after 24 hours of fasting were randomly selected from each tank and anesthetized to isolate posterior intestinal tissue for detection of gene expression, RNA-seq, miRNA-seq, and the activities of ATPase and Na^+^-K^+^-ATPase. The differentiation and separation of the posterior intestine referred to the method of Li et al. (28). The aforementioned samples were kept at −80°C until testing.

### 2.3 Enzyme activity determination

Posterior intestine tissue soaked in 9 volumes of phosphate-buffered saline (PBS) was disrupted and centrifuged to prepare tissue homogenate solution. It was used to determine glycinin contents with the plant glycinin ELISA kit (Jingmei Biological Technology Co., Ltd., Jiangsu, China), and the activities of ATPase and Na^+^-K^+^-ATPase were determined using the kit of Nanjing Jiancheng Bioengineering Institute (Nanjing, China; Cat. No. A070-1 and A070-2). Experimentation and computation were conducted following the instructions.

### 2.4 Real-time quantitative PCR

Detailed steps for real-time quantitative PCR (RT-qPCR) of inflammation-related mRNA and miRNA in the posterior intestine are provided in the **Supplementary Material**. Specific primers were designed according to the National Center for Biotechnology Information (NCBI) based on the yellow catfish gene sequences (**Supplementary Table S1**). The reference genes of mRNA and miRNA were *β-actin* (29) and U6 (30), respectively. The expression levels were quantitated by the 2^−ΔΔCT^ method (31).

### 2.5 RNA-seq and miRNA-seq analysis

The isolation, library construction, sequence determination, and function enrichment of RNA and miRNA were completed by Shanghai Meiji Biomedical Technology Co., Ltd. (Shanghai, China). Detailed instructions can be obtained from the **Supplementary Material**.

### 2.6 16S rRNA sequencing

Microbial 16S rRNA sequencing was conducted by Shanghai Personalbio Technology Co., Ltd. The specific steps can be obtained in the **Supplementary Material**.

### 2.7 Statistical analysis

Data were analyzed using the SPSS Statistics 26.0 software. The analysis methodology was similar to that of our previous study (27). In short, the normality distribution and variance homogeneity of data were examined using the Shapiro–Wilk and Levene’s tests, respectively. One-way ANOVA was employed to analyze the differences between different levels of glycinin groups. Multiple comparative analyses were executed by Tukey’s test. Linear and quadratic tendencies were assessed using orthogonal polynomial contrasts. Experimental data were presented as means ± standard error (means ± SE). Additionally, a difference was regarded as significant if *p* ≤ 0.05. Pearson’s correlation coefficient (PCC) was used to uncover the correlations between inflammatory cytokine gene expressions, the posterior intestinal microbiota abundance, and differentially expressed miRNA (DEmiRNA) expressions.

## 3 Results

### 3.1 Enzyme activities of posterior intestine

**Figure 1:**
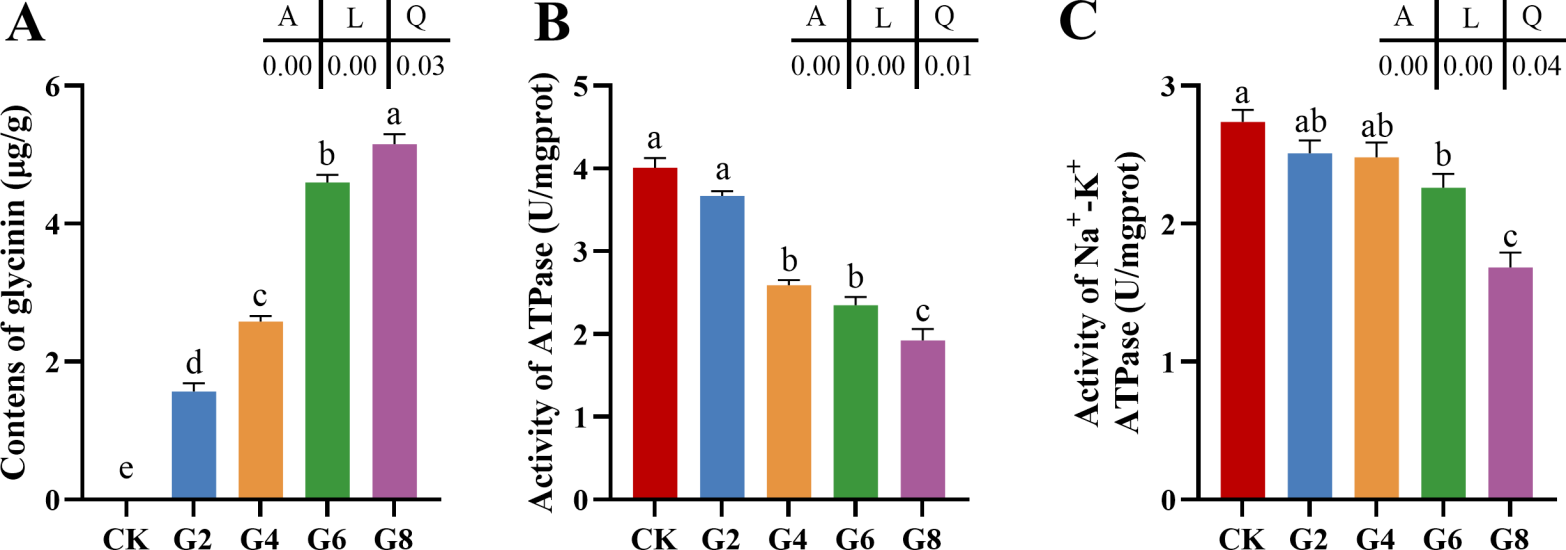
Glycinin contents **(A)** and the activities of ATPase **(B)** and Na^+^-K^+^-ATPase **(C)** in posterior intestine of *Pelteobagrus fulvidraco* ♀ × *Pelteobagrus vachelli* ♂. Obvious differences among groups are denoted by a, b, c, d, and e (*p* < 0.05). A indicates the *p*-value assayed via one-way ANOVA; L indicates a linear tendency assayed via orthogonal polynomial contrasts; Q indicates a quadratic tendency assayed via orthogonal polynomial contrasts.

With the rise of dietary glycinin level, the posterior intestinal glycinin content increased significantly and linearly (**Figure 1A**). In comparison to that in the CK group, ATPase activity was notably reduced in the G4, G6, and G8 groups (**Figure 1B**), and Na^+^-K^+^-ATPase activity was dramatically decreased in the G6 and G8 groups (**Figure 1C**). Additionally, significant linear and quadratic relationships were presented between the dietary glycinin levels and the activities of ATPase and Na^+^-K^+^-ATPase.

### 3.2 Relative expressions of inflammatory cytokine mRNAs in posterior intestine

**Figure 2:**
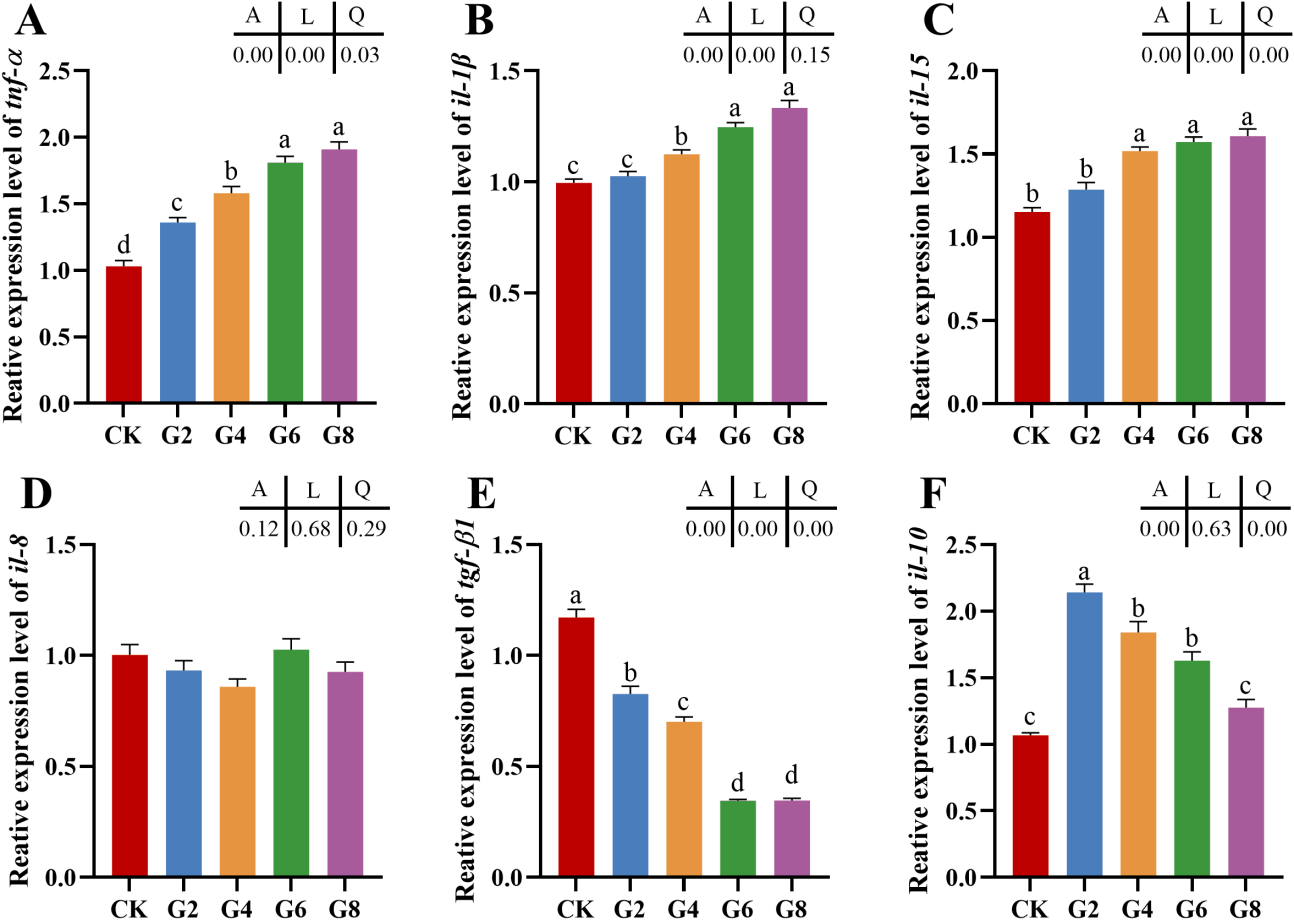
RT-qPCR of tnf-α **(A)**, il-1β **(B)**, il-15 **(C)**, il-8 **(D)**, tgf-β1 **(E)**, il-10 **(F)** mRNAs in posterior intestine of *Pelteobagrus fulvidraco* ♀ × *Pelteobagrus vachelli* ♂ (n = 3). The obvious differences among groups are denoted by a, b, c, and d (*p* < 0.05). A indicates the p-value assayed via one-way ANOVA; L indicates a linear tendency assayed via orthogonal polynomial contrasts; Q indicates a quadratic tendency assayed via orthogonal polynomial contrasts.

The impacts of glycinin in the diet on the mRNA expressions of inflammatory cytokines in the posterior intestine are presented in **Figure 2**. The expressions of *tumor necrosis factor* (*tnf-α*), *interleukin-1β* (*il-1β*), and *interleukin-15* (*il-15*) in the G4, G6, and G8 groups were considerably elevated in comparison to those in the CK group, but the expression of *transforming growth factor-β1* (*tgf-β1*) obviously dropped in the glycinin-added groups. Notably, the expressions of *tnf-α* and *il-1β* in the G6 and G8 groups were considerably elevated compared to those in the G4 group, while the *tgf-β1* expression was considerably reduced. Relative to that in the CK group, the expression of *interleukin-10* (*il-10*) was remarkably enhanced in the G2, G4, and G6 groups, with no obvious variation in the G8 group. Strong linear relationships were demonstrated among the levels of dietary glycinin and the expressions of *tnf-α*, *il-1β*, *il-15*, and *tgf-β1*. A notable quadratic trend was displayed in the expression of *il-10* with the increase of dietary glycinin levels. No considerable variation was noted in the expression of *interleukin-8* (*il-8*).

### 3.3 RNA-seq analysis of posterior intestine

**Figure 3:**
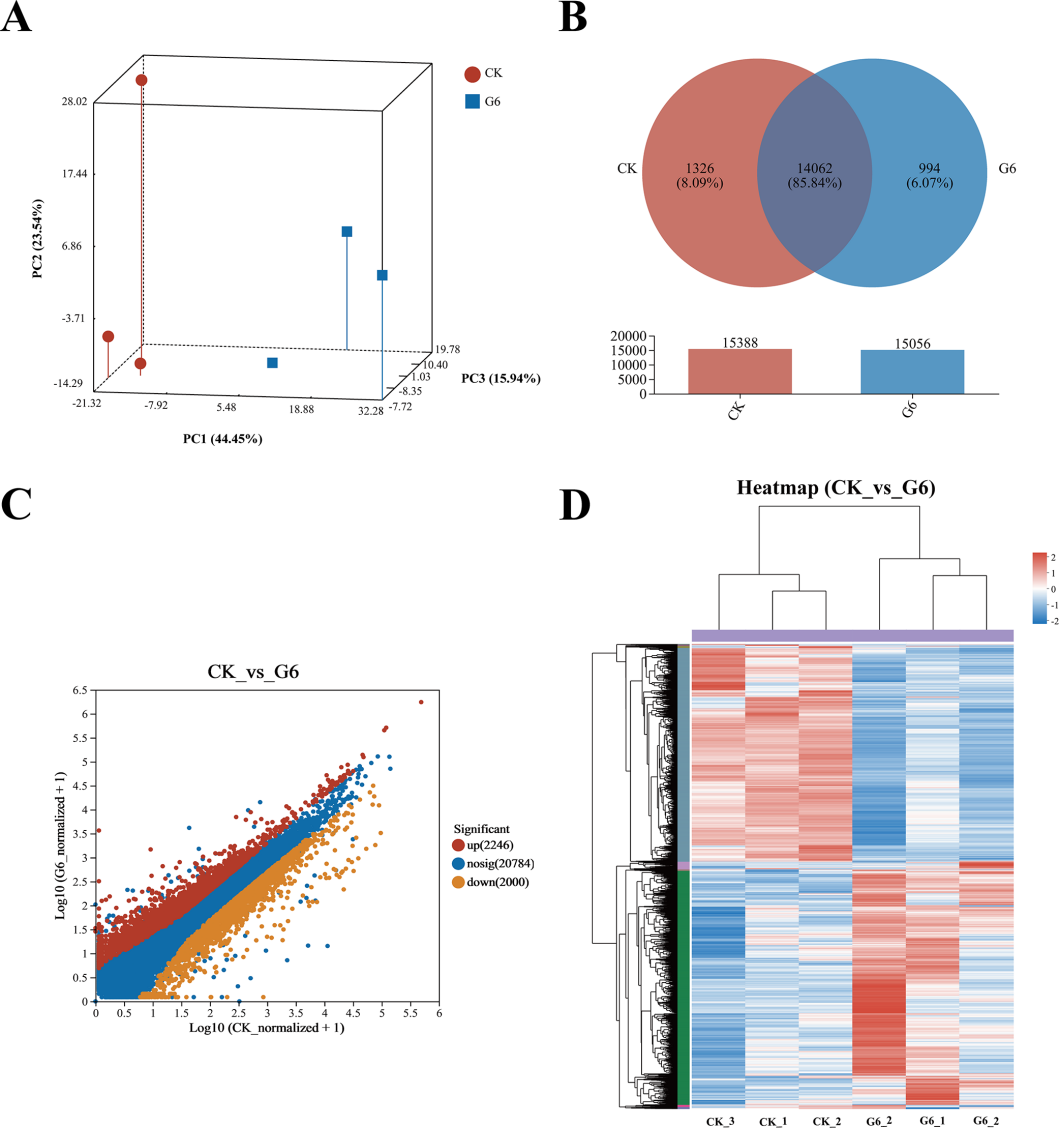
RNA-seq analysis of posterior intestine in the CK and G6 groups. **(A)** Principal component analysis (PCA). **(B)** Venn analysis. **(C)** Scatter diagram of differentially expressed genes (DEGs): red stands for upregulation, and yellow stands for downregulation (*p* < 0.05). **(D)** Cluster analysis of DEGs (heatmap): red indicates upregulation of DEGs, while blue represents downregulation.

Our previous study indicated that 5.45% feed glycinin significantly reduced the growth rate of hybrid yellow catfish and induced cell apoptosis (27), significantly reduced posterior intestinal ATPase and Na^+^-K^+^-ATPase activities, and induced the highest significance of pro-inflammatory factors level in this paper. RNA-seq was performed on the CK and G6 groups. A total of 38.75 Gb of high-quality data was acquired (**Supplementary Table S2**). Principal component analysis (PCA) suggested the variations in gene expression profiles among two groups (**Figure 3A**). Moreover, 8.09% and 6.07% of specific gene expressions were sequenced in the CK and G6 groups, respectively (**Figure 3B**). A total of 4,246 differentially expressed genes (DEGs) were obtained, with 2,246 upregulated and 2,000 downregulated genes (**Figure 3C**). In the cluster analysis, it was evident that a noticeable portion of the DEGs showed absolutely converse expression patterns between the CK and G6 groups (**Figure 3D**).

**Figure 4:**
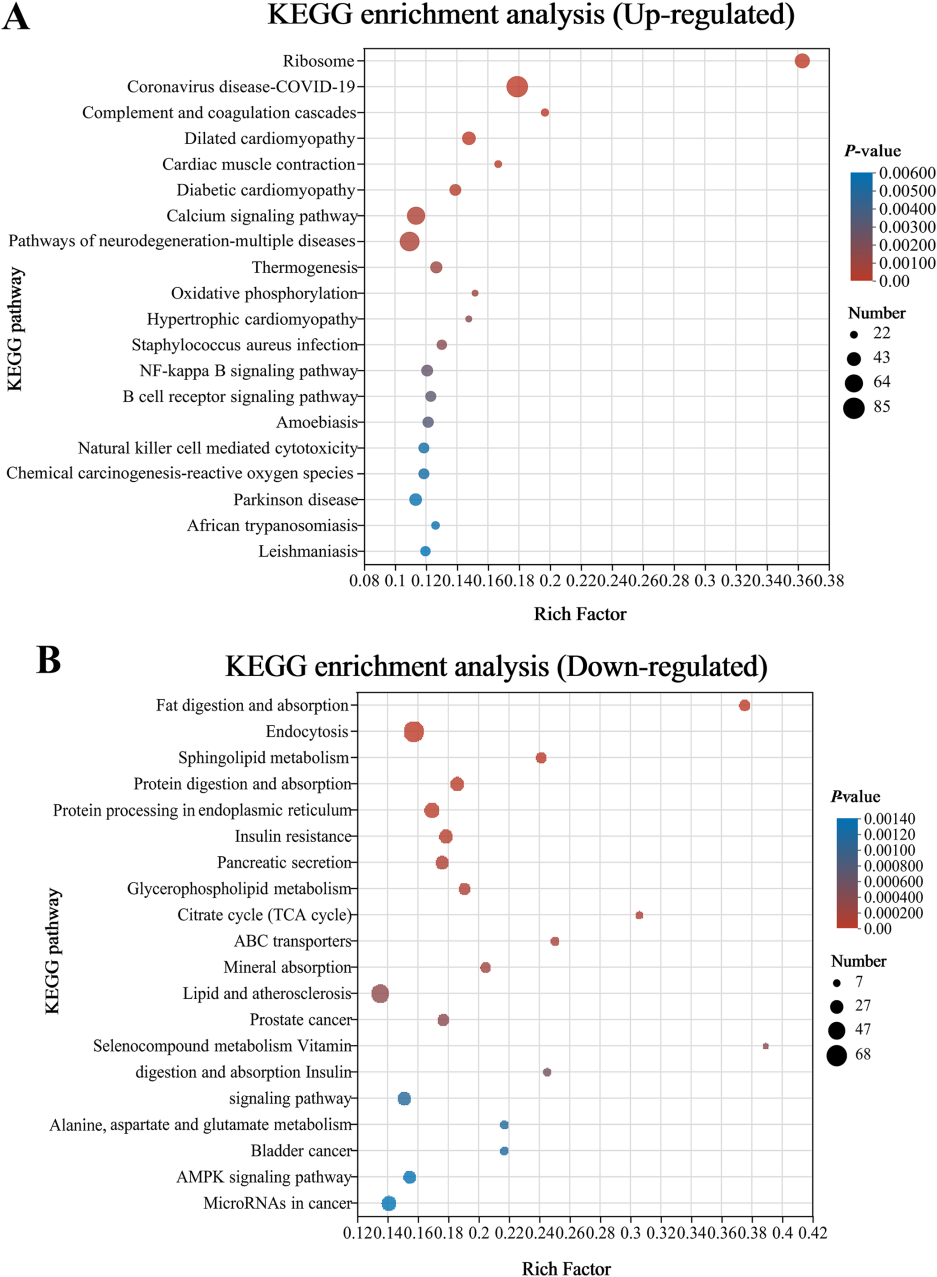
Bubble chart of top 20 Kyoto Encyclopedia of Genes and Genomes (KEGG) pathways concentrated with upward **(A)** and downward genes **(B)** in the CK and G6 groups. The more significant the rich factor, the higher the level of enrichment. The sizes of the dots represent the quantity of genes, and the colors of the dots are associated with various *p*-values. *p* < 0.05 is regarded as considerably enriched.

Analysis using the Kyoto Encyclopedia of Genes and Genomes (KEGG) disclosed that there was a considerable enrichment of elevated genes in 43 KEGG pathways. Remarkably enriched immune- and human disease-related pathways primarily included Coronavirus disease-COVID-19, Complement and coagulation cascades, *Staphylococcus aureus* infection, NF-kappa B (NF-κB) signaling pathway, B cell receptor signaling pathway, Natural killer cell mediated cytotoxicity, Fc epsilon RI signaling pathway, Inflammatory mediator regulation of TRP channels, MAPK (mitogen-activated protein kinase) signaling pathway, and Intestinal immune network for IgA production (**Figure 4A**; **Supplementary Table S3**). Additionally, the first 20 pathways obviously enriched by downregulated genes mainly involved nutrient absorption and metabolism, including Fat digestion and absorption, Endocytosis, Protein digestion and absorption, Protein processing in endoplasmic reticulum, Citrate cycle (TCA cycle), Glycerophospholipid metabolism, ABC transporters, Mineral absorption, Selenocompound metabolism, Alanine, aspartate and glutamate metabolism, and Vitamin digestion and absorption, as well as immune- and human disease-related pathways including Lipid and atherosclerosis, MicroRNAs in cancer, Focal adhesion, and Adherens junction (**Figure 4B**, **Supplementary Table S3**).

### 3.4 MiRNA-seq analysis of posterior intestine

**Figure 5:**
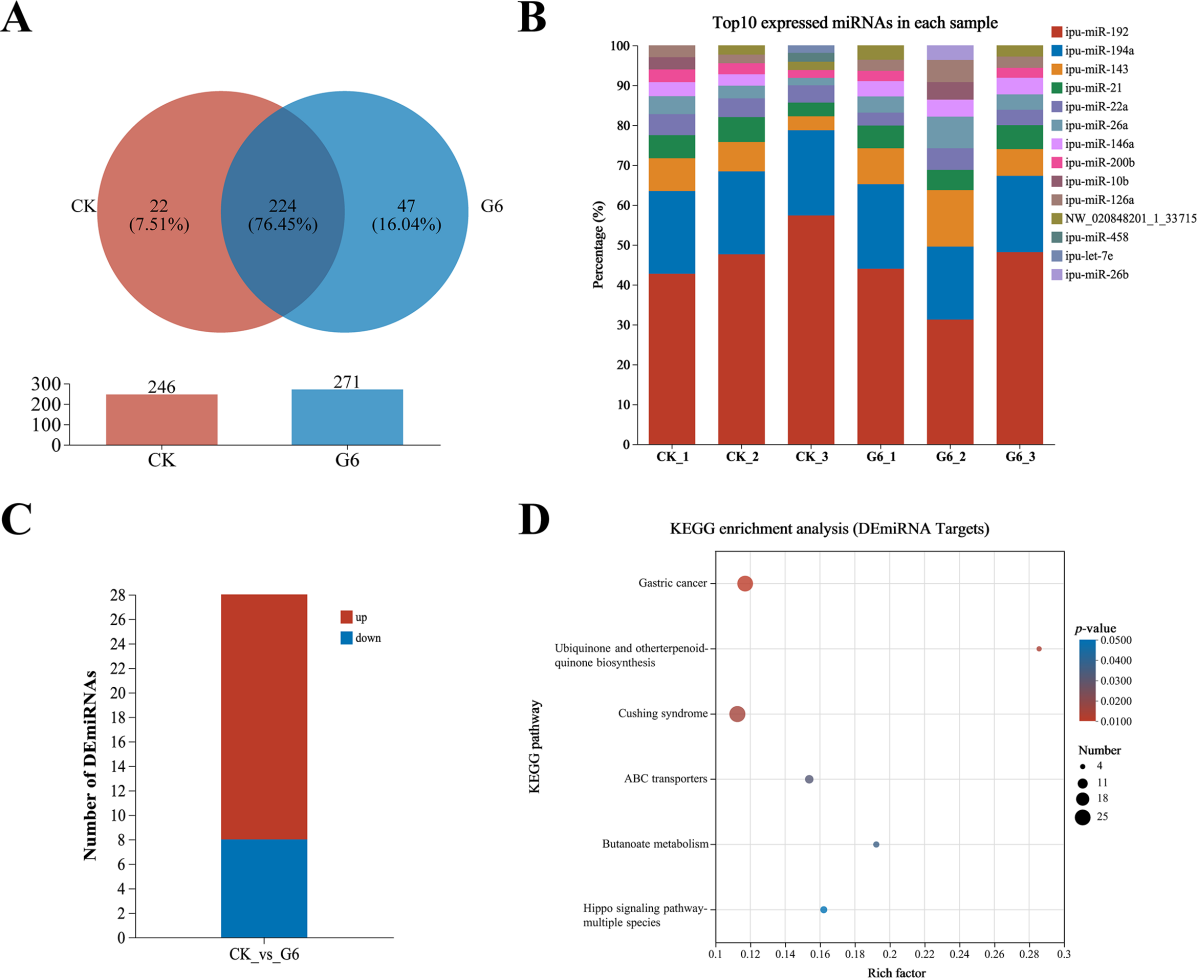
MiRNA-seq analysis of posterior intestine in the CK and G6 groups. **(A)** Venn analysis. **(B)** Top 10 expressed miRNAs in each sample. **(C)** Differentially expressed miRNAs (DEmiRNAs). **(D)** Top 20 Kyoto Encyclopedia of Genes and Genomes (KEGG) enrichment pathways of DEmiRNAs. Significant enrichment is indicated as −log10(*p*) > 0.

Six samples from the CK and G6 groups were sequenced with miRNA, and a total of 170 known miRNAs and 335 novel miRNAs were obtained (**Supplementary Table S4**). Analysis of miRNA expression results presented that 224 miRNAs were co-expressed between the CK and G6 groups, accounting for 76.45% of the total miRNAs, and the unique miRNAs of the G6 group were 8.53% higher than those of the CK group (**Figure 5A**). The top 10 expressed miRNAs in different samples were ipu-miR-192, ipu-miR-194a, ipu-miR-143, ipu-miR-21, ipu-miR-146a, ipu-miR-22a, ipu-miR-26a, ipu-miR-126a, NW_020848201_1_3371, ipu-miR-200b, ipu-miR-10b, ipu-miR-26b, ipu-miR-458, and ipu-let-7e (**Figure 5B**). DEmiRNAs were screened by differential expression analysis, with the involvement of 20 upregulated and eight downregulated genes (**Figure 5C**). Among the seven known DEmiRNAs discovered, ipu-miR-216b, ipu-miR-29c, ipu-miR-216a, ipu-miR-217, ipu-miR-184, and ipu-miR-459 were significantly reduced, and ipu-miR-489 was significantly enhanced (**Supplementary Table S4**).

A total of 15,466 target genes were predicted using miRanda and RNAhybrid software, including 1,736 DEmiRNA targets. DEmiRNA targets were enriched into 108 KEGG pathways (**Supplementary Table S5**), of which significantly enriched immune- and human disease-related pathways included Cushing syndrome and Gastric cancer (**Figure 5D**). Interestingly, the signaling pathways mainly involved in DEmiRNA targets were almost identical to those involved in DEGs in the aforesaid immune pathways, such as MAPK, WNT, PI3K-AKT, and TGF-β (**Supplementary Table S5**).

### 3.5 DEmiRNAs targeting DEGs

**Figure 6:**
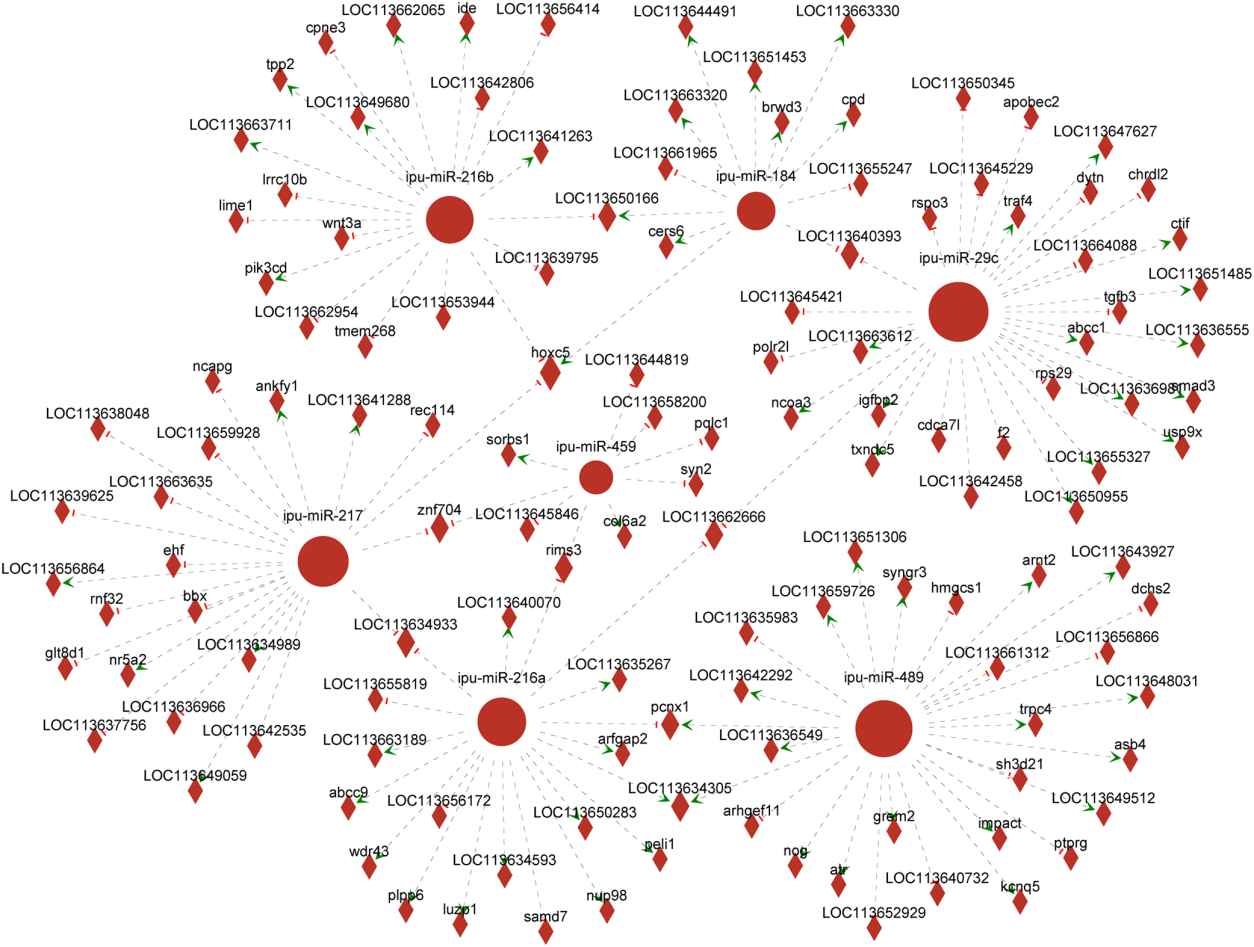
Network analysis of the association between known differentially expressed miRNAs (DEmiRNAs) and their targeting differentially expressed genes (DEGs). Circular nodes represent known DEmiRNAs; diamond nodes represent target genes; short red vertical bars represent negative regulation, and green arrows represent positive correlation.

Association analysis of DEmiRNAs and targeting DEGs revealed 366 DEmiRNA–DEG interactions. We selected known DEmiRNAs to construct their interactome with DEGs (**Figure 6**) and screened for DEmiRNA–DEG pairs associated with inflammation. For instance, miRNA-216b could negatively regulate the expression of *wnt3a* (|PCCs| = 0.66, *p* = 0.16). Ipu-miR-29c may be the regulator of *smad3* (PCCs = 1, *p* = 0.00), *traf4* (PCCs = 0.88, *p* = 0.02), *txndc5* (PCCs = 0.83, *p* = 0.04), etc. Additionally, ipu-miR-217 could negatively direct the expression of *ehf* (|PCCs| = 0.83, *p* = 0.04).

### 3.6 RT-qPCR verification of DEGs and DEmiRNAs

**Figure 7:**
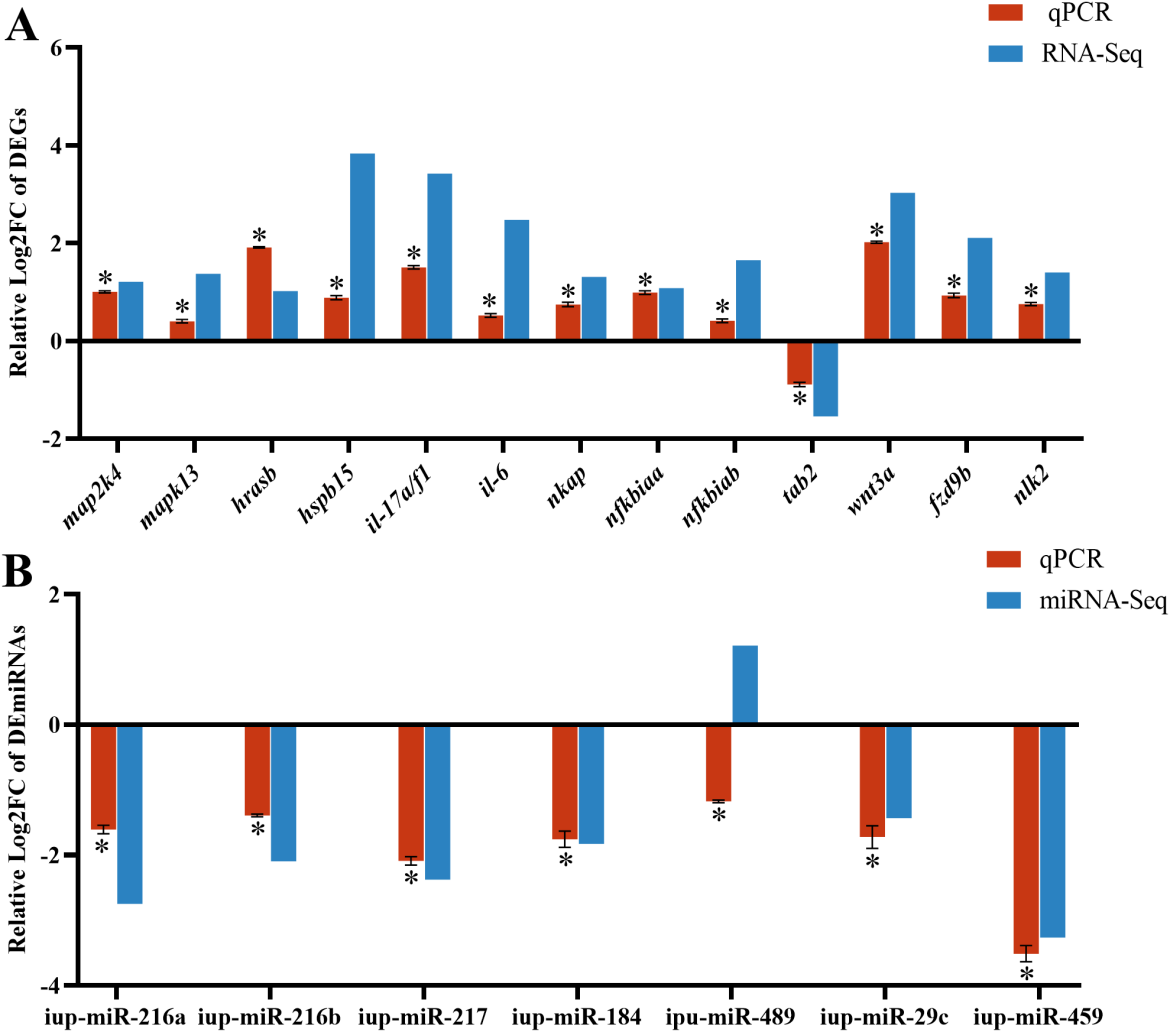
RT-qPCR results of differentially expressed genes (DEGs) **(A)** and differentially expressed miRNAs (DEmiRNAs) **(B)** in the CK and G6 groups. Data describe mean ± SE (n = 3). Relative log2Fold Chang (log2FC) reveals the changes between two groups of samples in expression levels and normalized by change in the reference gene of mRNA (*β-actin*) or miRNA (U6). Asterisks indicate obvious differences in DEGs or DEmiRNAs validated by qPCR between two groups of samples (*p* < 0.05).

To confirm the reliability of RNA-seq and miRNA-seq, the DEG expressions associated with the MAPK, NF-κB, and WNT pathways and known DEmiRNAs were validated by RT-qPCR. Our findings exhibited almost identical expression patterns between RT-qPCR results of DEmiRNAs and DEGs and their respective transcriptome sequencing data, except for up-miR-489 (**Figure 7**).

### 3.7 Microbial analysis of posterior intestine

**Figure 8:**
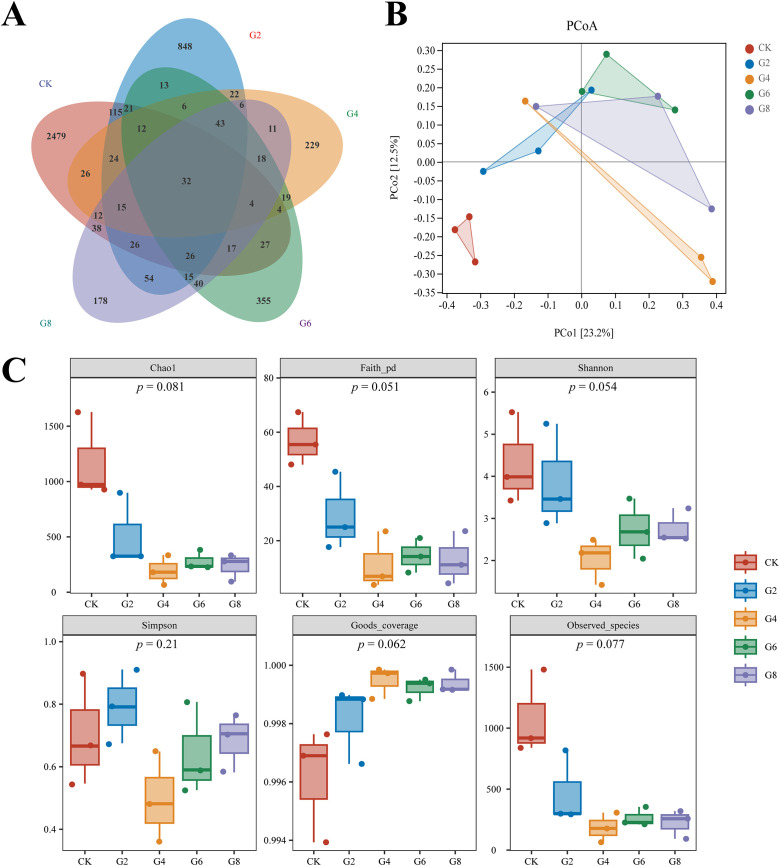
Microbial diversity in posterior intestine of *Pelteobagrus fulvidraco* ♀ × *Pelteobagrus vachelli* ♂. **(A)** Venn analysis. **(B)** Principal coordinate analysis (PCoA). **(C)** Alpha-diversity analysis.

Inflammation may contribute to the loss of intestinal microbiota balance; similarly, dysregulation of the microbiota may directly or indirectly affect the development of inflammation and immune-mediated pathologies. Thus, 16S rRNA sequencing was conducted in all groups to analyze the microbial composition of the posterior intestine. Our data presented that the highest number of 2,479 unique operational taxonomic units (OTUs) was observed in the CK group; interestingly, a reduced number of OTUs was presented in the glycinin-added groups (**Figure 8A**). There were 115 most shared OTUs between the G2 and CK groups. Principal coordinate analysis (PCoA) based on Unweighted_uniFrac further presented that the coordinates of the CK group were separated from the glycinin-added groups (**Figure 8B**). Alpha-diversity analysis suggested that there were no considerable differences between groups (**Figure 8C**). However, compared with those in the CK group, the indices such as Chao 1, Faith_pd, Shannon, and Observed_species displayed a downtrend in the glycinin-added groups (**Figure 8C**).

**Figure 9:**
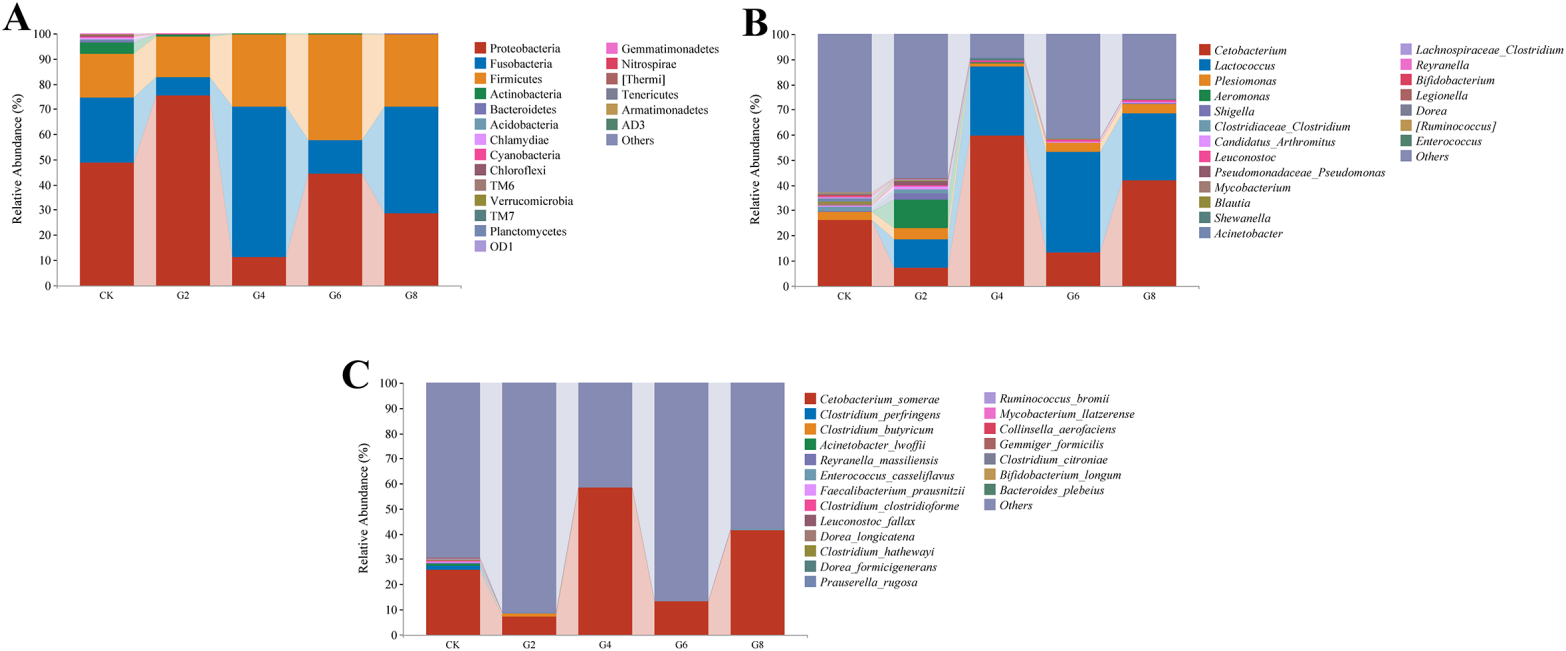
Microbial composition in posterior intestine of *Pelteobagrus fulvidraco* ♀ × *Pelteobagrus vachelli* ♂. **(A)** Phylum level. **(B)** Genus level. **(C)** Species level.

Microbial composition analysis showed that Proteobacteria (41.50% ± 8.05%), Fusobacteria (29.76% ± 8.74%), and Firmicutes (26.73% ± 7.81%) were identified as the predominant bacterial phyla of the posterior intestine from all groups (**Figure 9A**; **Supplementary Table S6**). *Cetobacterium* (29.56% ± 8.71%) had the highest abundance in all groups. However, the subdominant genera were different in different groups, with *Plesiomonas* (3.46% ± 1.18%) and Clostridiaceae *Clostridium* (1.87% ± 0.77%) in the CK group; *Lactococcus* (11.32% ± 1.86%), *Aeromonas* (11.41% ± 8.90%), and *Plesiomonas* (4.28% ± 2.69%) in the G2 group; and *Lactococcus* (31.33% ± 11.01%) and *Plesiomonas* (2.74% ± 0.85%) in the G4, G6, and G8 groups (**Figure 9B**; **Supplementary Table S6**). The average abundance at the species level was mainly dominated by *Cetobacterium somerae* (29.02% ± 8.54%) (**Figure 9C**; **Supplementary Table S6**).

**Figure 10:**
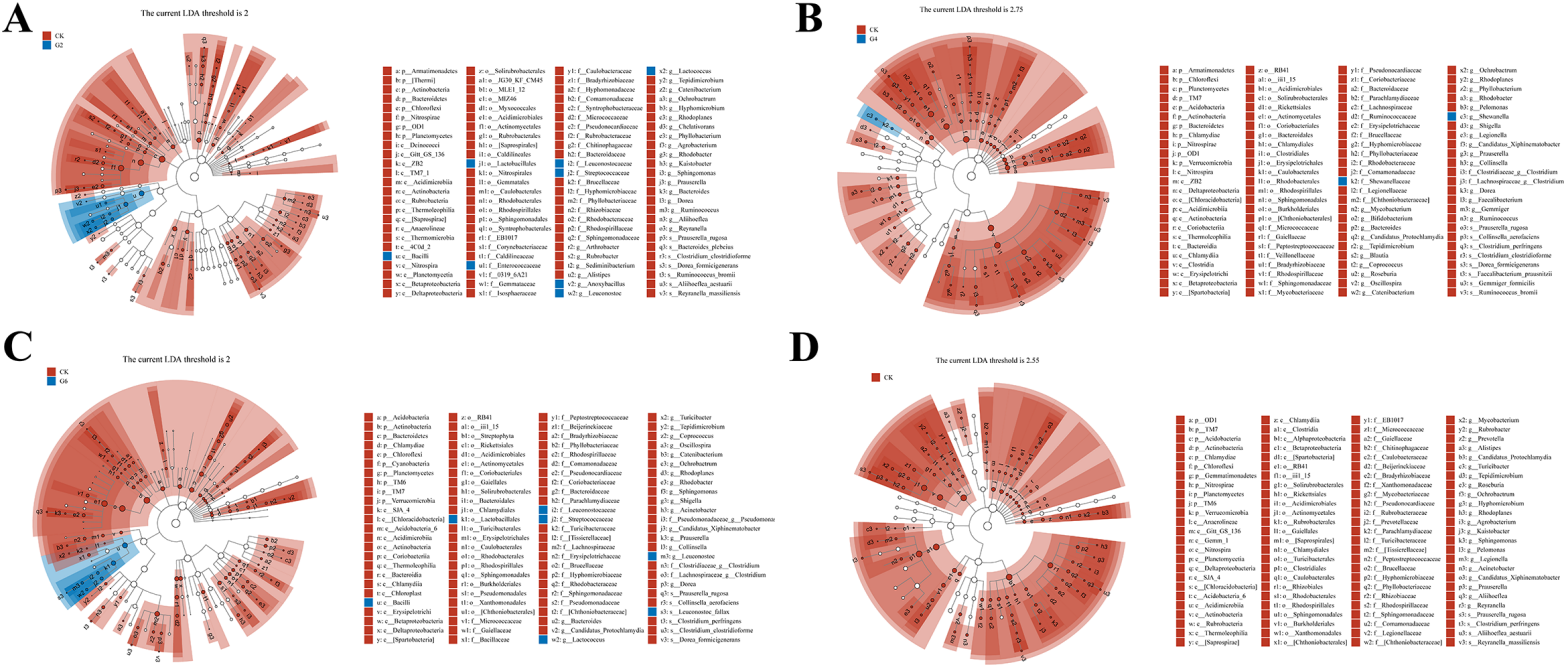
Linear discriminant analysis (LDA) effect size (LEfSe) analysis of posterior intestinal microbes in *Pelteobagrus fulvidraco* ♀ × *Pelteobagrus vachelli* ♂. **(A)** CK vs. G2. **(B)** CK vs. G4. **(C)** CK vs. G6. **(D)** CK vs. G8. Blue or red nodes represent a notable rise in the abundance of the group represented by that color. Letters indicate the name of the flora or species with significant differences between groups. LEfSe scores of the significantly different microbiota are higher than the LDA threshold.

Linear discriminant analysis effect size (LEfSe) was performed to identify the steady difference in species between different groups (**Figure 10**). The results indicated that the phylum level abundances including Actinobacteria, Bacteroidetes, Acidobacteria, Chloroflexi, Chlamydia, Cyanobacteria, and TM6 presented significant reductions in the glycinin-added groups relative to the CK group. At the species level, based on the presence of a certain level of glycinin in this experimental diet, the relative abundances of *Prauserella rugosa*, *Ruminococcus bromii*, *Bacteroides plebeius*, *Faecalibacterium prausnitzii*, *Reyranella massiliensis*, *Aliihoeflea aestuarii*, *Collinsella aerofaciens*, *Dorea formicigenerans*, *Clostridium perfringens*, *Clostridium clostridioforme*, and *Gemmiger formicilis* were downward significantly comparable with those in the CK group. It is worth mentioning that the abundance of *Leuconostoc fallax* in the G6 group was remarkably upward compared to that in the CK group.

**Figure 11:**
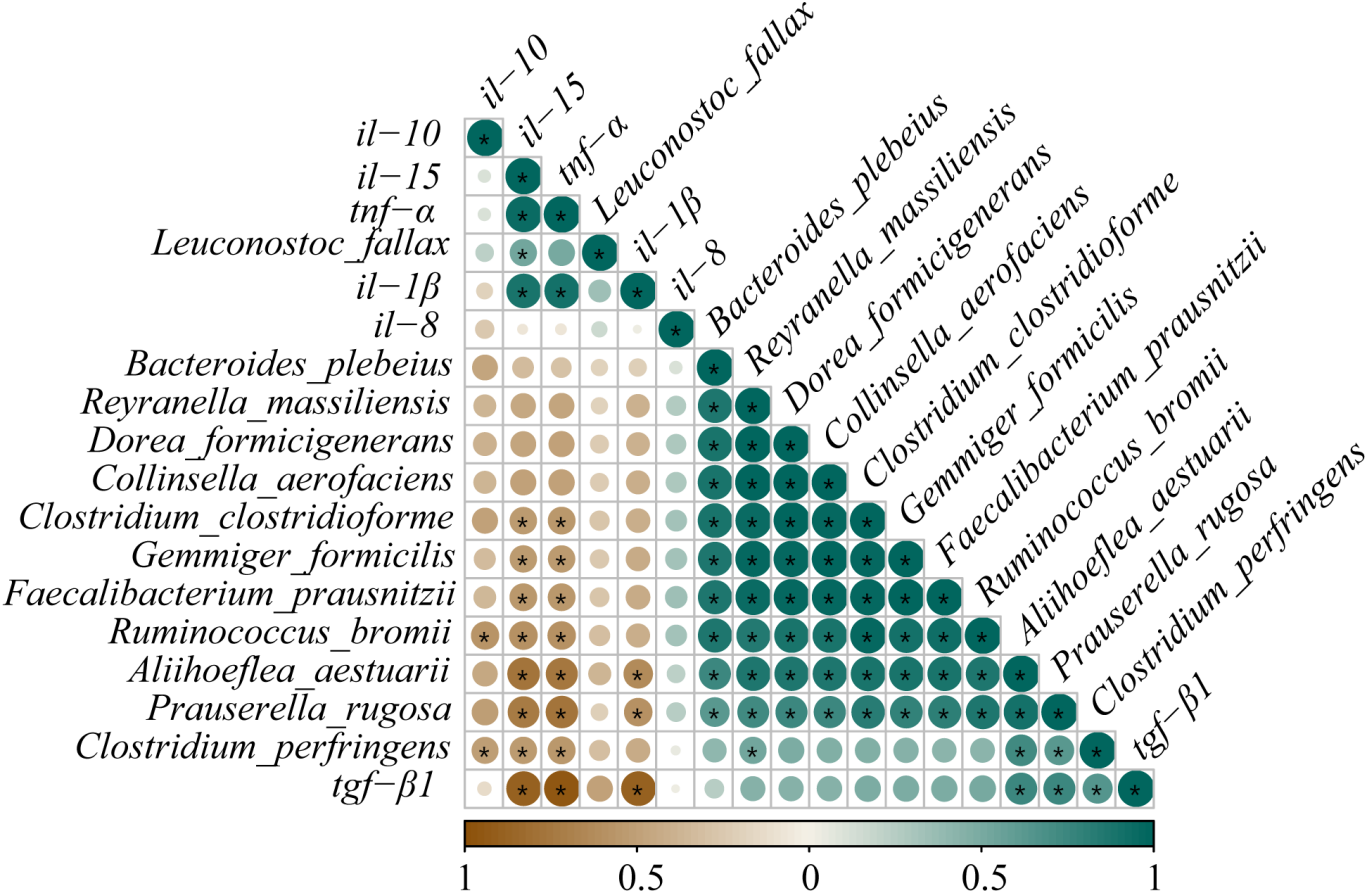
Correlation analysis between differential species microbes and the expressions of inflammatory cytokine mRNAs in posterior intestine of *Pelteobagrus fulvidraco* ♀ × *Pelteobagrus vachelli* ♂. Brown indicates negative correlation, and green indicates positive correlation. The size of circle is positively correlated with Pearson’s correlation coefficient (PPC). Asterisks indicate notable distinctions (*p* < 0.05).

Pearson’s correlation analysis was employed to estimate the association between the expressions of inflammatory cytokine mRNAs and the abundances of differential species microbes (**Figure 11**). Significantly negative correlations were discovered between the abundances of *R. bromii* and *C. perfringens* and the *il-10* expression (0.57 ≥ |PCCs| ≥ 0.52). Similarly, the abundance changes in *P. rugosa*, *R. bromii*, *F. prausnitzii*, *A. aestuarii*, *C. perfringens*, *C. clostridioforme*, and *G. formicilis* presented noticeable negative correlations with the *tnf-α* and *il-15* expressions (0.78 ≥ |PCCs| ≥ 0.53). The abundances of *P. rugosa* and *A. aestuarii* showed an eminent opposite connection with the *il-1β* expression (0.63 ≥ PCCs ≥ 0.58). Contrarily, remarkable positive connections were displayed between the abundances of *A. aestuarii*, *P. rugosa*, and *C. perfringens* and the *tgf-β1* expression (0.75 ≥ PCCs ≥ 0.65). The *L. fallax* abundance and the *il-15* expression also showed an obvious positive relationship (PCCs = 0.53). The aforementioned results confirmed that dietary glycinin levels were associated with microbial modulation that was in turn associated with induced inflammation.

The KEGG pathway function annotation displayed that the microbial functional potential is primarily enriched in material metabolism, such as amino acids, carbohydrates, cofactors, and vitamins (**Supplementary Figure S1A**). Subsequently, MetagenomeSeq was performed to identify the metabolic pathways with significant differences between the CK group and the glycinin-added groups (**Supplementary Figure S1**, **Supplementary Table S7**). Results showed that relative to those in the CK group, 2, 16, 19, and 17 significant metabolic pathways were observed in the G2, G4, G6, and G8 groups, respectively. The *S. aureus* infection pathway was noticeably enhanced in the G2 and G6 groups (**Supplementary Figures S1B, D**). Nevertheless, more downregulated differential pathways were screened out in the G4, G6, and G8 groups, including Lysosome, Atrazine degradation, Cyanoamino acid metabolism, Proteasome, Polyketide sugar unit biosynthesis, NOD-like receptor signaling pathway, Protein digestion and absorption, Apoptosis, and Steroid biosynthesis (**Supplementary Figures S1C–E**).

### 3.8 Correlation analysis of DEmiRNAs and microbiota

**Figure 12:**
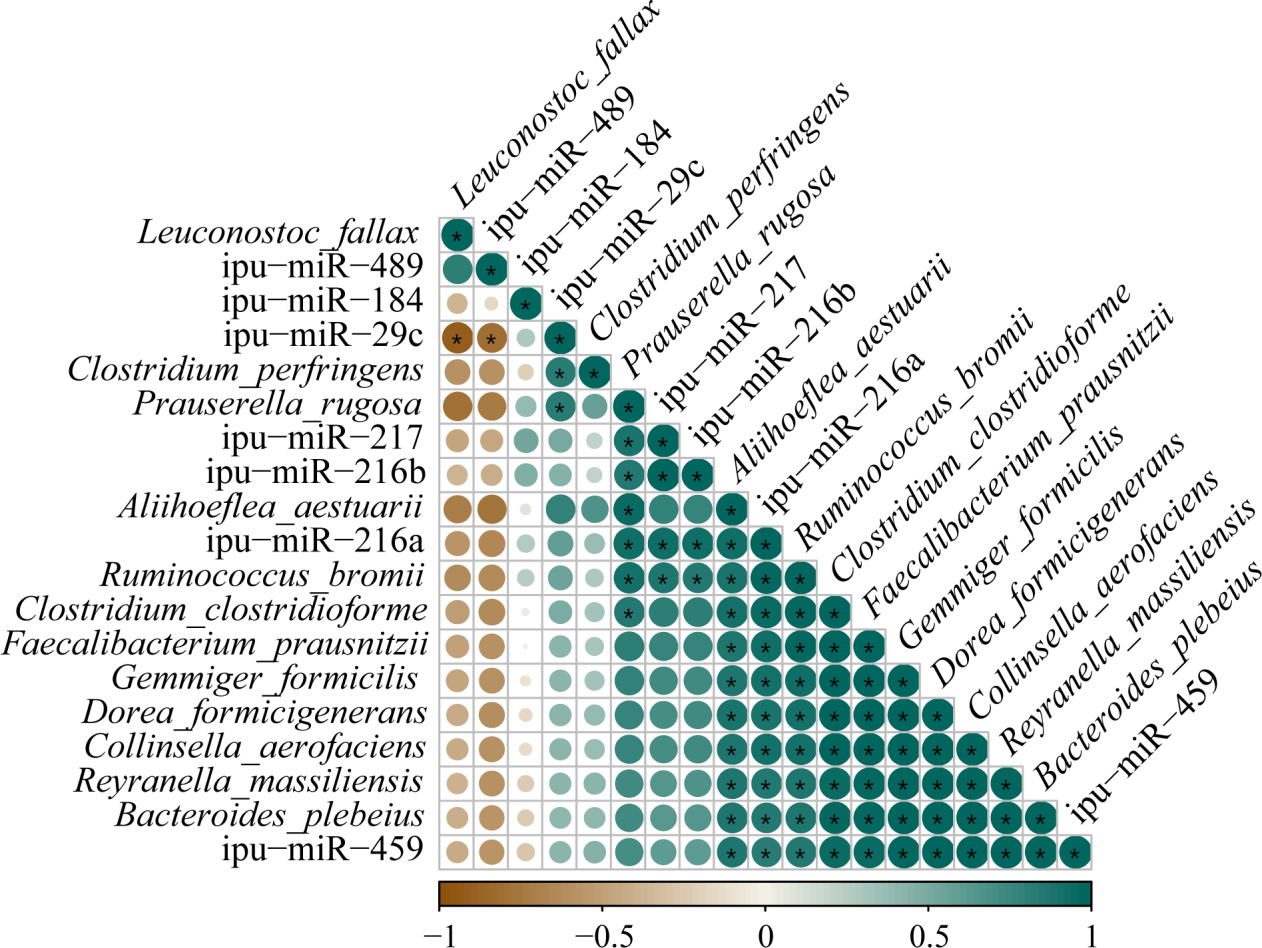
Correlation analysis between the expressions of known differentially expressed miRNAs (DEmiRNAs) and the abundances of differential species microbes in posterior intestine of *Pelteobagrus fulvidraco* ♀ × *Pelteobagrus vachelli* ♂. Brown indicates negative correlation, and green indicates positive correlation. The size of circle is positively correlated with Pearson’s correlation coefficients (PPCs). Asterisks indicate notable distinctions (*p* < 0.05).

Correlations between the expressions of DEmiRNAs and the abundances of differential species microbes in the posterior intestine are shown in **Figure 12**. The ipu-miR-29c expression showed a robust negative correlation with the *L. fallax* abundance (|PCCs| = 0.90); contrastingly, it was eminently positively correlated with the abundances of *P. rugosa* and *C. perfringens* (0.83 ≥ PCCs ≥ 0.82). Additionally, noticeable positive relationships were observed between the expressions of ipu-miR-217 and ipu-miR-216b and the abundances of *P. rugosa* and *R. bromii* (0.88 ≥ PCCs ≥ 0.85). Furthermore, there were dramatic positive correlations between the expressions of ipu-miR-216a and ipu-miR-459 and the abundances of *R. bromii*, *B. plebeius*, *F. prausnitzii*, *R. massiliensis*, *A. aestuarii*, *C. aerofaciens*, *D. formicigenerans*, *C. clostridioforme*, and *G. formicilis* (0.99 ≥ PCCs ≥ 0.83).

## 4 Discussion

### 4.1 High levels of dietary glycinin reduced the activities of ATPase and Na^+^-K^+^-ATPase in posterior intestine

Homeostasis of fish intestinal function is an important defense line against exogenous antigen invasion. As a foreign antigen with strong immunogenicity, a small portion of glycinin that is difficult to enzymatically hydrolyze would maintain macromolecular activity and directly cross the intestinal barrier, stimulating the immune response of blood, lymph, and intestinal mucosa to generate intestinal dysfunction (12, 32). In this study, a significant positive correlation was observed between the glycinin contents in the posterior intestine and the levels of dietary glycinin, indicating that glycinin may exist in the form of macromolecules to damage the intestine. It should be emphasized that specific IgM against glycinin was notably increased in the blood of turbot that was given 8.31% dietary glycinin (7), suggesting that glycinin induced an immune response in fish.

To further explore the effect of dietary glycine on the absorption of intestinal nutrients in hybrid catfish, we examined the activities of ATPase and Na^+^-K^+^-ATPase in this study. ATPases are a type of membrane proteins that play an essential role in the transport of substances during cellular metabolism (33). Na^+^-K^+^-ATPase creates a beneficial transcellular Na gradient that is essential for the effective operation of Na-dependent nutrient co-transporters on the brush border membrane of intestinal epithelial cells and can indirectly indicate the absorptive ability of intestinal mucosa (34, 35). Our assay results presented that the activities of ATPase and Na^+^-K^+^-ATPase were dramatically diminished linearly with the increase of the dietary glycinin levels, suggesting that glycinin may interrupt the absorption of nutrients. The consistent results have also been reported in a study of mirror carp (*Cyprinus carpio*) (36). Consistently, RNA-seq results revealed that pathways associated with nutrient absorption and metabolism were obviously enriched by downregulated genes, including those related to fats, proteins, glycerophospholipids, minerals, amino acids, and vitamins. Furthermore, our earlier study indicated that high levels of dietary glycinin considerably reduced the protein efficiency ratio of feed and the crude lipid content of whole fish. These findings supported the additional lines of evidence that glycinin may influence the nutrient transport and absorption capabilities of enterocytes. It is notable that inhibited Na^+^-K^+^-ATPase activity in the inflamed mucosa was attributed to the elevation of specific inflammatory mediators (37). Similarly, the diminishment of Na^+^-K^+^-ATPase in chronic enteritis of rabbits inhibited the brush border membrane Na-glucose cotransport pathway (38).

### 4.2 High levels of dietary glycinin disturb the expressions of inflammatory cytokine mRNAs in posterior intestine

Glycinin entering the blood and lymph stimulated the intestinal mucosa to produce an immunologic response and cause changes in cytokines (8, 32). Cytokines of the intestine are central players in the regulation of immunological responses after mucosal insults and the dominance of homeostatic or inflammatory conditions (39). In a previous study, we noticed that the integrity of posterior intestinal conformation was impacted by 3.57% glycinin, while 5.45% and 7.27% dietary glycinin groups were more serious (27). Our results of this paper validated that the expressions of the pro-inflammatory genes (*tnf-α* and *il-1β*) and immunoregulatory gene (*il-15*) increased dramatically at 3.57% or higher dietary glycinin levels, whereas the expression of anti-inflammatory cytokine *tgf-β1* was depressed by dietary glycinin, suggesting that excessive dietary glycinin may induce the occurrence of enteritis in hybrid yellow catfish. These were consistent with the research outcomes on golden crucian carp (*C. carpio* × *Carassius auratus*) (3), grass carp (12), hybrid grouper (*Epinephelus fuscoguttatus* ♀ × *Epinephelus lanceolatus* ♂) (32), and *R. lagowskii* Dybowski (6). During inflammation, immune cells rush into the intestinal mucosa, affecting the function of epithelial cells by producing IL-1 and TNF-α (40). Meanwhile, T lymphocytes mediate inflammatory suppression by secreting cytokines (IL-10 and TGF-β) to maintain intestinal homeostasis (41). IL-15, an immunoregulatory cytokine with multiple functions, evokes improved innate immunity to shape adaptive immunity, which is accompanied by the rise of its level following pathogenic encounters with the host (42). Furthermore, a prior study has shown that the administration of IL-15 results in the development of severe inflammatory arthritis (43).

Interestingly, the *il-10* expression was considerably elevated in the G2, G4, and G6 groups in this research, while no notable distinction was observed in the CK and G8 groups. Activated monocytes and lymphocytes secrete IL-10 to inhibit the formation of pro-inflammatory cytokines (IL-1, IL-6, TNF-α, etc.); otherwise, the balance of pro- and anti-inflammatory systems may be disrupted with the continuous increase of pro-inflammatory cytokines, which may exacerbate the inflammation (44). Correspondingly, the high expression of *il-10* mRNA under the stimulation of dietary glycinin may be an adaptive mechanism for the body to suppress the persistence and severity of inflammation, while 7.27% dietary glycinin may severely destroy the immune system homeostasis. Inconsistently, high levels of dietary glycinin induced an attenuated *il-10* expression in the posterior intestine of turbot (7) and *C. carpio* var. Jian (45), which may be associated with the stage of the body’s inflammatory resistance mechanism or different exposure times to glycinin.

### 4.3 RNA-seq analysis of posterior intestine

To obtain insights into the underlying mechanisms of enteritis activated by glycinin, RNA-seq identified 4,246 DEGs between the CK and G6 groups in the posterior intestine of hybrid yellow catfish. As is well known, under the stimulation of exogenous heat sources, inflammatory neurotransmitters are released, activating immune cells and causing sustained tissue damage or repair. In this study, numerous upregulated DEGs by dietary glycinin were eminently enriched in the immune cell response-related KEGG pathways such as Complement and coagulation cascades, B cell receptor signaling pathway, Natural killer cell mediated cytotoxicity, Inflammatory mediator regulation of TRP channels, and Intestinal immune network for IgA production. Foreign antigens activate myeloid leukocytes, which are then degraded by leukocytes and presented to T cells (46). Subsequently, CD4+ T cells polarize and secrete special cytokines to execute immune function (47). As in higher vertebrates, natural killer (NK)-like cells and CD8+ T cells enable the elimination of infected cells and protect the host from severe damage (48). DEGs also were partly amassed in the T-cell receptor signaling pathway, Th1 and Th2 cell differentiation, and Th17 cell differentiation in this study (**Supplementary Table S2**), suggesting that glycinin activates the cellular immune response of fish intestine. B-cell receptors recognize pathogenic signals and trigger a series of intricate biological responses to play a role in humoral defense (49). Antigen–antibody binding activates the complement system, promoting processes such as inflammation and apoptosis (50). The interplay of complement and coagulation is crucial in the development and treatment of inflammation, which also involves the modulation of inflammatory cytokines (51). TRP channels in immune cells mediate the production and release of inflammatory mediators (52). The intestinal immune network for IgA production contributes to maintaining a peaceful bacteria–host interaction (53). Similarly, the response of the aforementioned pathways to pathogens has been pointed in fish such as *P. vachelli* (54), hybrid grouper (55), and Mandarin fish (*Siniperca chuatsi*) (56). It is a wonder that *S. aureus* infection was found in both the enrichment pathway of DEGs and the prediction of microbial functional potential, revealing that glycinin may increase the susceptibility of the fish intestine to pathogenic bacteria.

Differently, downregulated DEGs were noticeably enriched in the nutrient metabolism such as proteins, lipids, and amino acids. Significantly, downregulated microbial functional pathways in the G4, G6, and G8 groups were also clustered in metabolism including proteins and steroids. Consistently, our prior study validated that 6% or higher dietary glycinin significantly reduced feed protein conversion efficiency and the crude lipid content in entire fish and muscle of experimental fish (27), suggesting that glycinin not only caused the immunoreaction of the body but also led to the disorder of nutrient metabolism. Similar results were observed in enteritis induced by soybean meal in hybrid grouper (55).

Furthermore, the co-enrichment of DEGs and DEmiRNA target genes yielded intriguing results as the significant responses of the MAPK, NF-κB, and WNT pathways in glycinin-induced enteritis. MAPK and NF-κB, as classical signaling pathways of inflammation (57), have been declared on their response mechanism to glycinin in piglets (58), grass carp (12), *C. carpio* (59), etc. IL-17A/F1 promotes the expressions of *il-1β*, *tnf-α*, *il-6*, chemokines, and antibacterial peptides and activates the MAPK and NF-κB signal pathways in fish (60). Consistently, the validation of RT-qPCR indicated that the expressions of MAPK (*map2k4*, *mapk13*, *hrasb*, and *hspb15*) and NF-κB (*il-17a/f1*, *il-6*, *nakp*, *nfkbiaa*, and *nfkbiab*) pathway-related genes were upregulated by 5.45% dietary glycinin in hybrid yellow catfish intestine. Intriguingly, MAPK13 is one of the four p38 MAPKs that exert its function acting on pro-inflammatory signaling (61). As a subfamily of the MAPK superfamily, the p38 MAPK is essential in the inflammatory stress response (62), activated by various growth factors, inflammatory cytokines, or a range of environmental pressures (63). The p38 MAPK has been previously identified as being able to elevate the approachability of the hidden NF-κB binding sites to activate the NF-κB pathway (64). A recent study reported that hypoxic–ischemic conditions induce inflammation and enhance cytotoxicity through the p38MAPK/NF-κB pathways in microglial cells (65). Additionally, the present study demonstrated that glycinin exposure stimulated the WNT signaling pathway, involving the immunomodulation of inflammation characterized by increased *wnt3a*, *fzd9b*, and *nlk2* mRNA expressions. Indeed, the WNT pathway and inflammatory signaling cascades interact significantly with each other (66). Wnt signaling is essential for modulating the immune system by controlling inflammatory cytokines including NF-κB and its associated genes (*il-6*, *il-8*, and *tnf-*α) (67) and manipulating the proliferation and differentiation of intestinal epithelial cells under inflammation conditions (66). Ayers et al. (68) reported that suppressing WNT signaling alleviates cholestatic injury by destroying the NF-κB-dependent inflammatory axis. Notably, past studies have suggested that WNT can synergistically drive tumorigenesis with ERK signaling (69) and can also cooperate with p38 MAPK to regulate cell proliferation (70). Nevertheless, further research is required to validate the significance of these findings in relation to inflammatory diseases.

### 4.4 MiRNA-seq analysis of posterior intestine

The role of miRNA has been considered in inflammation (71). It is well known that miRNA disturbances may affect multiple cellular pathways, as a single miRNA can control several target genes simultaneously. Lines of evidence suggest that numerous miRNAs, such as miR-192, miR-143, miR-21, miR-146a, miR-26a/26b, miR-126, and miR-200b, contribute to protecting or destroying the tight junctions of the intestine and involve the process of inflammation by inhibiting or stimulating immune cells such as T cells, neutrophils, macrophages, or monocytes (72). Apparently, abundant expressions of these miRNAs were also found in this study. Furthermore, our study suggested that 5.45% dietary glycinin noticeably attenuated the known miRNA expressions of ipu-miR-216a, ipu-miR-216b, ipu-miR-217, ipu-miR-184, ipu-miR-29c, and ipu-miR-459, showing that high levels of dietary glycinin may disrupt host miRNA expression patterns. Related studies have reported that the overexpression of miR-216a can inhibit lipopolysaccharide (LPS)-induced cell apoptosis and autophagy and regulate JAK2-STAT3 and NF-κB signal transduction to reduce inflammatory damage (73). MiR-216b has been extensively studied and identified as a regulator of inflammation as well as a factor that suppresses tumors (74). Additionally, miR-217 knockdown aggravated the inflammation in the endothelial cells of the human aorta, increasing IL-16, IL-β, and TNF-α levels (75). Similarly, miR-184 and miR-29c potentially participate in the signal transduction related to inflammation and apoptosis, and their overexpression suppressed pro-inflammatory cytokine release (IL-16, IL-β, etc.) in microglial inflammation triggered by LPS (76, 77). These results suggest that excessive feed glycinin may regulate the occurrence and development of intestinal inflammation by inhibiting inflammation-related miRNAs.

Correlation network analysis displayed that DEmiRNAs may regulate the inflammation-related genes to participate in glycinin-induced enteritis. Recent research indicated that hsa−miR−216a−3p manipulates cell hyperplasia in oral cancer through Wnt3a/β−catenin signaling (78). In our study, the expression of *wnt3a* may be regulated by ipu-miR-216b. Additionally, the *smad3* gene controls inflammatory cell recruitment through TGF-β signaling (79). Earlier research about Crohn’s disease showed that TRAF4 represses the stimulation of NF-κB caused by NOD2 (80). TXNDC5 plays a role in initiating and progressing inflammation (81). The mRNA expression of the aforementioned regulatory factors may be regulated by ipu-miR-29c in glycinin-induced enteritis. Additionally, EHF is crucial for preserving the balance of epidermal and colonic epithelial tissues (82), and this gene may be regulated by ipu-miR-217 in this research. However, the specific miRNAs targeting regulatory relationships mentioned above need to be further studied.

### 4.5 Microbial analysis of posterior intestine

A plethora of studies have revealed a connection between intestinal microflora dysbiosis and inflammation (83–85). Exploring the response mechanisms of intestinal microbiota to glycinin-induced enteritis is a key process for understanding intestinal health. The diversity and stability of intestinal microbiota are closely related to fish diseases (86). In the present study, the alpha diversity of microflora in the posterior intestine was not eminently affected by dietary glycinin, but the richness indices (Chao 1 and Observed_species), the evolutionary diversity index (Faith_pd), and the number of OTUs showed a certain decreasing trend in the glycinin-added groups, especially in the high-level glycinin groups. In comparison to healthy fish, lower alpha diversity was detected in diseased fish including salmon (*Plecoglossus altivelis*) (86), yellow catfish (*P. fulvidraco*) (28), and grouper (*E. coioides*) (87). Microbial diversity may be reduced by high levels of glycinin to induce dysbiosis and inflammation. Previous research has reported that the active antimicrobial peptide segments produced by glycinin decomposition can inactivate microbial cells through cell membrane pores or membrane permeability (88).

In order to discover bacteria susceptible to glycinin, we explored phyla displaying significant reactions to dietary glycinin regardless of dosage. The results revealed that Proteobacteria, Fusobacteria, and Firmicutes were the main phyla, which is consistent with the core microbiota found in the intestine of *P. fulvidraco* (89), grass carp (90), and common carp (*C. carpio* L.) (91). It is noteworthy that relative abundances of Actinobacteria, Bacteroidetes, Acidobacteria, Chloroflexi, Chlamydia, Cyanobacteria, and TM6 significantly dwindled after adding a certain level of dietary glycinin in this experiment, which may be a specific manifestation of reduced microbial community richness by dietary glycinin. A study on Chinese mitten crab claimed that 80 g/kg dietary glycinin considerably increased the abundances of Actinobacteria and Proteobacteria in the intestine, while the abundance of Bacteroidetes was decreased (8). Nonetheless, the overall structure of intestinal microbiota was not significantly affected by dietary glycinin in turbot (7). These inconsistent results may be partly associated with differences in dietary glycinin levels, species, or aquaculture environment.

Correspondingly, the levels of dietary glycinin changed to varying degrees in the dominant microbiota at the genus level. *Cetobacterium* was identified as a superior genus in each group, with the representative being *C. somerae*. The metabolism of *C. somerae* produces acetic acid, which promotes insulin expression and glycometabolism and plays an important regulatory role in fish health (92). Clostridiaceae encompassing *Clostridium* appeared in higher abundance in the feces of dogs fed meat diets, indicating its potential correlation with protein digestibility (93). Intriguingly, both microbial function prediction and RNA-seq validated that the protein digestion and absorption pathway was significantly downregulated by dietary glycinin, which may be related to the attenuation of *Clostridium* abundance. The stimulation of *Leuconostoc* abundance in the G6 group should be noted. The inflammatory cases of *Leuconostoc* infection have been continuously reported (94, 95), and they are associated with potential and frequent gastrointestinal diseases (95).

Similar to the phylum level, most microbial species were significantly inhibited by the dietary glycinin of different levels, which further indicated that glycinin causes disruption of intestinal microbiota. Among them, *R. bromii*, *F. prausnitzii*, *C. clostridioforme*, and *B. plebeius* are potential probiotics, which produce secondary metabolites with strong biological activity such as short-chain fatty acids, mainly in the antibacterial, anti-tumor, or anti-inflammatory role, and protect the intestinal mucosal barrier from damage by foreign antigens (96–98). In addition, *R. massiliensis* was considered to enhance the stress resistance of tropical gars (*Atractosteus tropicus*) in adverse environments (99). A recent study also found the absence of most microflora, including *D. formicigenerans*, in Saudi inflammatory bowel disease (IBD0 patients (100). In contrast, *C. perfringens*, a conditioned pathogen secreting virulent toxins, triggers related intestinal diseases (101). There is relatively little research on *P. rugosa*, *A. aestuarii*, *C. aerofaciens*, and *G. formicilis*, particularly in the intestine of fish. All aforesaid results demonstrated that the ecological balance of microbial communities may be disrupted by dietary glycinin by inhibiting the intestinal microbiota reproduction of hybrid yellow catfish, especially potential probiotics.

Correlation analysis addressed the noticeable negative relationships among the remarkably different bacteria and the mRNA expressions of *tnf-α*, *il-1β*, and *il-15*. Khan et al. (102) found that *F. prausnitzii* inhibits the secretion of IL-8 activated by IL-1β in Caco-2 cells. In rheumatoid arthritis, with higher IL-6 levels, attenuated *R. bromii* was identified by machine learning (103). Additionally, the possible cause of inhibited *C. perfringens* could be an increase in *il-10* expression. A study in chicken revealed *il-10* production in necrotic enteritis induced by *C. perfringens* (104). These findings suggest that the reduced intestinal microbiota may be related to the occurrence of enteritis, but whether inflammatory cytokines are the direct factors that inhibit or facilitate the growth of intestinal microbes needs to be studied more comprehensively.

### 4.6 Correlation analysis of DEmiRNAs and microbiota

Past studies have revealed that host-derived miRNAs are vital for maintaining normal intestinal microbiota (21, 23). MiR-21, an important factor involved in the pathogenesis of intestinal inflammation, aggravated dextran sodium sulfate (DSS)-induced colitis in mice by affecting the intestinal microflora (105). Released miRNA is taken up by bacteria, e.g., *Fusobacterium nucleatum* and *Escherichia coli*, and combines with DNA to specifically manipulate gene expression and, consequently, alter microbial adaptability (24). Additionally, it is important to highlight that miRNA may indirectly affect microbial dysbiosis by regulating the production of cytokines (106). MiR-21 activates macrophages and naive CD4+ cells to release inflammatory mediators, intensifying colitis, which may indirectly influence the homeostasis of the microbiota (107).

Intriguingly enough, host miRNA expression is also affected by intestinal microbiota (108). A study demonstrated that probiotics including *Lactobacillus fermentum* and *Lactobacillus salivarius* restored the miRNA expression such as miR-143 in mice with DSS-induced colitis (109). Additionally, a certain study reported a strongly positive relationship between *Faecalibacterium* and hsa-miR-6833-5p (25). In our study, *F. prausnitzii* presented significant positive correlations with the expressions of ipu-miR-216a and ipu-miR-459. However, so far, there is no literature exploring the direct relationship between the microbes and the DEmiRNAs of this study. The microbiota may affect host miRNA expression through metabolites (e.g., short-chain fatty acids), bacterial endotoxins, and other factors, thereby influencing the host’s intestinal health (110, 111). Anzola et al. (112) deciphered that bacterial component LPS via TLR4/MyD88/NF-κB-AKT enhances miR-146a levels in non-tumoral rat ileal cells to affect inflammation. Nonetheless, the exact interaction mechanism between specific miRNAs and microorganisms still needs further research.

## 5 Conclusion

Taken together, dietary 5.45% or higher levels of glycinin induced enteritis through the MAPK/NF-κB/WNT pathway, and inflammatory state could disrupt micro-ecology balance by host-secreted miRNA in hybrid yellow catfish. Otherwise, the observations in this study regarding the partial immune regulatory network of intestinal mRNA, miRNA, and microbes are based on the transcriptional profiles obtained and should be further verified in future studies by *in vitro* experiments, luciferase assay, or fecal microbiota transplant experiments.

## Data Availability

The data of Transcriptome and Small RNA sequencing are deposited in the NCBI SRA database. The names of the repository/repositories and accession number(s) can be found below: https://www.ncbi.nlm.nih.gov/, PRJNA1207723; https://www.ncbi.nlm.nih.gov/, PRJNA1207605. And the other data can be found in the link: https://www.jianguoyun.com/p/DdZdUyQQ1JKXDRiFl-cFIAA.
