# Supplementary material for: Integrative RNA, miRNA, and 16S rRNA sequencing reveals immune-related regulation network for glycinin-induced enteritis in hybrid yellow catfish, Pelteobagrus fulvidraco ♀ × Pelteobagrus vachelli ♂
Source: Front Immunol. 2025 Jan 15;15:1475195. doi: 10.3389/fimmu.2024.1475195 (PMC11775447; doi:10.3389/fimmu.2024.1475195)
Supplement: Supplementary file 1 [file Table1.docx]

Supplementary Material

# Supplementary Methods and Materials

## Real-Time Quantitative PCR (RT-qPCR)

The total RNA of posterior intestine was extracted by RNA extract solution (Cat. No. G3013, Servicebio, China). A Nanodrop 2000 spectrophotometer was used to determine the purity and quality of total RNA at a ratio of 260 / 280 nm. Referring to the instructions, RNA was reverse transcribed with the SweScript RT I First Strand cDNA Synthesis Kit (Servicebio, China). The kit of 2×SYBR Green qPCR Master Mix (None ROX) (Servicebio, China) was utilized for RT-qPCR with the QuantStudio^TM^ 6 Flex System (Life technologies, USA). MiRNA reverse transcription was conducted with the miRNA first-strand cDNA synthesis kit (Cat. No. G3334, Servicebio, China). Subsequently, 2×Universal Blue SYBR Green qPCR Master Mix (Cat. No. G3326, Servicebio, China) was subjected to qPCR detection. The 20 μl reaction system was carried out on 96-well plastic plate and each sample was repeated three time. Specific primers were designed with NCBI based on the yellow catfish gene sequences, and miRNA primers were designed by miRNA Design V1.01 software (Supplementary Table S1). The expressions of mRNA and miRNA were quantitated by the 2^–ΔΔCT^ method.

## RNA-seq of posterior intestine

The isolation, library construction, sequence determination and function enrichment of mRNA were completed by Shanghai Meiji Biomedical Technology Co., Ltd. Briefly, total RNA was extracted of posterior intestine from the CK and G6 groups (TRIzol® Reagent, Invitrogen, USA), and mRNA was enriched by A-T base pairing with poly A using magnetic beads with Oligo (dT). After the mRNA was fragmented, under the action of reverse transcriptase, random hexamers primers were added to reverse transcription to synthesize cDNA. Cohesive ends of cDNA were modified into blunt ends with end Repair Mix, and connected the adaptor. The library was created by Truseq^TM^ RNA sample prep Kit (Illumina, USA). Subsequently, PCR quantification by carried out with the QuantiFluor® dsDNA System (Promega, USA), and the product was sequenced on the Illumina Novaseq 6000 platform (Illumina, USA). Based on the statistics of base distribution and quality fluctuation of each cycle of all sequenced reads, clean reads were obtained and then compared with the reference genome. RSEM (Version 1.3.3) was utilized for quantitative analysis of the gene expressions. The quantitative indicator was Transcripts Per Million (TPM), that is, the reading segment from a transcript in each million reading segments. The considerable expression of read counts was executed by DESeq2 (Version 1.10.1). The default threshold for variance analysis was restricted to *p* < 0.05 & |log2Fold Change (log2FC)| ≥ 1, and the up / down variance multiple set as 2. Besides, the multiplex test was corrected with the Benjamini & Hochber method. The enrichment analysis of KEGG (Version 2017.08) pathways was conducted by Goatools (Version 0.6.5), and *p* < 0.05 was regard as a considerable enrichment. Parameters without special instructions were the default values.

## MiRNA-seq of posterior intestine

The isolation, library construction, and high throughput sequencing of miRNA were completed by Shanghai Meiji Biomedical Technology Co., Ltd. Total RNA extraction was performed from the posterior intestine of the CK and G6 groups using Trizol Reagent (Invitrogen, USA). The Truseq^TM^ Small RNA sample prep Kit (Illumina, USA) was utilized to connect the 3' and 5' end joints, synthetize cDNA and construct the library. PCR amplification was performed with the QuantiFluor® dsDNA System (Promega, USA) and the amplified products were sequenced on the Illumina sequencing platform (USA). Clean reads were obtained by using Fastp (Version 0.19.5), and the known miRNAs annotation information were obtained by comparing reads with miRbase (Version Release 22) and Rfam (Version Rfam v12.3) database. MiRNA expression levels were determined by miRDeep2 (Version 2.0.1.3), and estimated by TPM. The miRNA target genes were predicted by Miranda (Version v3.3a). The considerable expressions of read counts and the functional enrichment of target genes were the same as transcriptome gene analysis method.

## 16S rRNA sequencing in posterior intestine

The microbial 16S rDNA gene amplicon sequencing was conducted by Shanghai Personalbio Technology Co., Ltd. Following the manufacturer's guidelines, the posterior intestinal contents from all groups were extracted microbial DNA with the OMEGA Soil DNA Kit (D5625-01) (Omega Bio-Tek, USA). The quantitative and qualitative assessment of DNA were executed by the NanoDrop 2000 UV-vis spectrophotometer (Thermo Scientific, USA). Through the thermocycler PCR system (GeneAmp 9700, USA), used primers 338F (5'-ACTCCTACGGGAGGCAGCA-3') and 806R (5'-GGACTACHVGGGTWTCT AAT-3') to amplify the V3-V4 hypervariable region in the 16S rRNA gene of microorganisms. The purification of PCR amplicons was carried by Vazyme VAHTSTM DNA Clean Beads (Vazyme, China) and quantitated by the Quant-iT PicoGreen dsDNA Assay Kit (Invitrogen, USA) on the Microplatereader (BioTek, FLx800). The library was constructed utilizing the TruSeq Nano DNA LT Library Prep Kit (Illumina, USA). Purified amplicons were sequenced on the Illumina MiSeq platform with MiSeq Reagent Kit v3 (illumina, USA). Bioinformatics for the microbiome were conducted using QIIME2 (v2019.4) ([1](#A1Bolyen2019)) following the official tutorials (<https://docs.qiime2.org/2019.4/tutorials/>). Concisely, the DADA2 method was used to remove primers, mass filter, denoise, splicing, and de-chimera of the original sequence ([2](#A2Callahan2016)). Then effective sequences were grouped into OTUs based on 97% similarity with Vsearch (v2.13.4_linux_x86_64). OTUs were classified by the ribosomal database project classifer soft. Alpha- and Beta-diversity were evaluated by the QIIME2 (v2019.4) software. Additionally, under the one-against-all comparison strategy, LDA Effect Size (LEfSe) was employed to examine species differences. Microbial functional potential predicted by PICRUSt2 software.

# Supplementary Figures

**Supplementary Figure 1.** KEGG metabolic pathways **(A)** and differences analysis **(BCDE)** of posterior intestinal microbiota in hybrid yellow catfish. In the difference analysis chart, Y-axis is the KEGG pathway; X-axis is log2FC, the positive value of log2FC represents an up-regulation in the glycinin-added group compared to the CK group, while the negative value represents a downregulation. Different colors represent the degree of significance. **(B)** CK vs G2; **(C)** CK vs G4; **(D)** CK vs G6; **(E)** CK vs G8.

# References

1. Bolyen E, Rideout JR, Dillon MR, Bokulich NA, Abnet CC, Al-Ghalith GA, et al. Reproducible, interactive, scalable and extensible microbiome data science using QIIME 2. *Nat Biotechnol*. (2019) 37(8):852-857. doi: 10.1038/s41587-019-0209-9
2. Callahan BJ, McMurdie PJ, Rosen MJ, Han AW, Johnson AJ, Holmes SP. DADA2: High-resolution sample inference from Illumina amplicon data. *Nat Methods*. (2016) 13(7):581-3. doi: 10.1038/nmeth.3869
